# A Case of Trauma-Induced Arterial Thrombosis Mimicking Vasculitis

**DOI:** 10.7759/cureus.10576

**Published:** 2020-09-21

**Authors:** Tanureet Kochar, Khalil Bourji, Housam Sarakbi

**Affiliations:** 1 Sleep Medicine, Wayne State University Detroit Medical Center, Detroit, USA; 2 Rheumatology, Wayne State University Detroit Medical Center, Detroit, USA

**Keywords:** vasculitis, trauma, vasculitis mimickers, thrombosis, arterial

## Abstract

Vasculitis mimickers are structural or pathologic entities that resemble the vasculitis clinical presentation and/or diagnostic findings. Their presence can be a conundrum, and physicians require careful assessment and adequate knowledge physicians when considering a diagnosis of vasculitis. Although they are considered mimickers, the therapeutic approach for most of them differs widely from that of vasculitis as high-dose steroids and potent immunosuppressive regimens are usually indicated in the latter. In fact, steroid therapy is contraindicated and is considered harmful in some of these mimickers (e.g. segmental arterial mediolysis). Therefore, it is important to distinguish them from vasculitis to prevent complications from immunosuppressive therapy. Hereby, we present a challenging case of a 64-year-old man who presented with acute gangrenous changes on his right fingers due to arterial thrombus after trauma resembling vasculitis.

## Introduction

Vasculitis has a wide range of presentations, from only cutaneous manifestation to multisystem organ involvement. There are many medical conditions that mimic vasculitis and have been known to cause a vasculitis-like picture. These include infections, drugs, and malignancy [1]. Miloslavsky et al. stated the following nine medical conditions as challenging primary vasculitis mimickers: fibromuscular dysplasia, calciphylaxis, segmental arterial mediolysis, antiphospholipid syndrome, hypereosinophilic syndrome, lymphomatoid granulomatosis, malignant atrophic papulosis, livedoid vasculopathy, and immunoglobulin G4-related disease [2]. Their presence can be a conundrum, and physicians require careful assessment and adequate knowledge when considering a diagnosis of vasculitis. Although they are considered mimickers, the therapeutic approach for most of them differs widely from that of vasculitis, as high-dose steroid and potent immunosuppressive regimens are usually indicated in the latter. In fact, steroid therapy is contraindicated and is considered harmful in some of these mimickers (e.g. segmental arterial mediolysis) [3]. Arterial thrombus developing after trauma can present as gangrene in the distal territory of the affected artery. The acuity and the clinical manifestation of arterial thrombosis can mimic vasculitis. It is prudent to distinguish the vasculitis mimickers from true vasculitis to prevent unnecessary immunosuppressive therapy, which has its own complications [1-2,4].

## Case presentation

**Figure 1:**
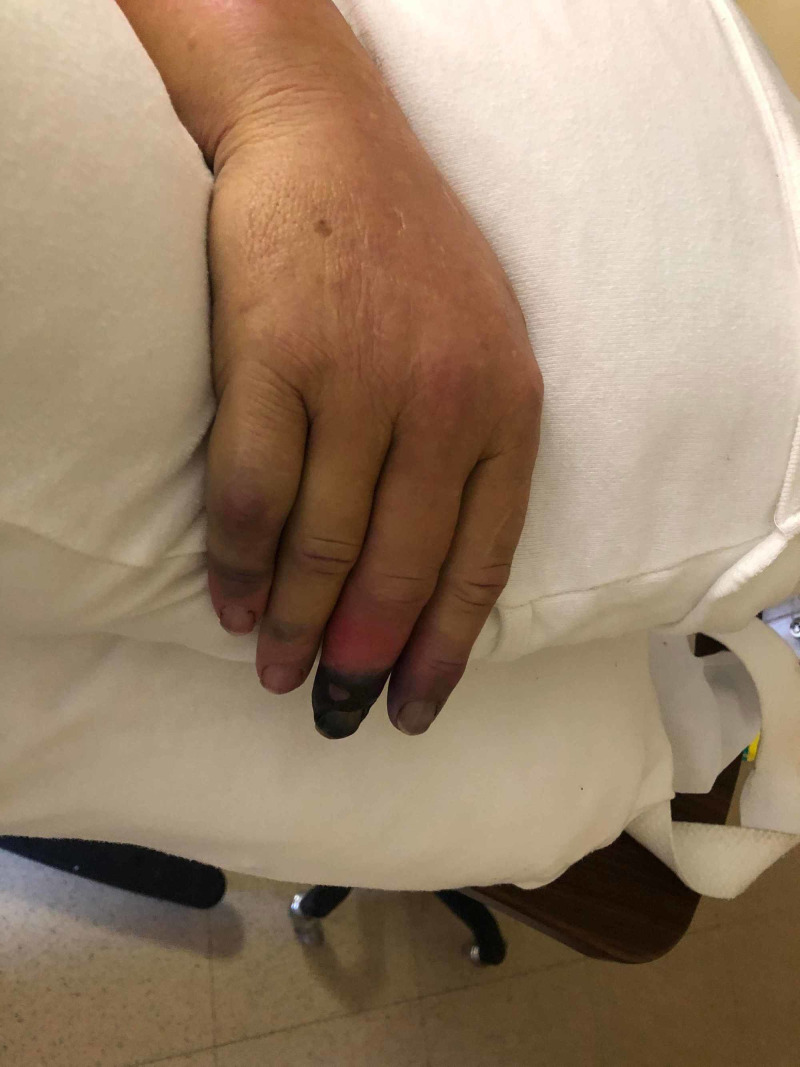
Gangrene at the right middle finger

A 64-year-old Caucasian man with a past medical history of hypertension with an unremarkable history of cardiovascular diseases or other major risk factors (tobacco use, diabetes, dyslipidemia, or family history), presented to the emergency department (ED) with pain and cold sensation in the second, third, and fourth fingers of his right hand. He reported that he was chopping a tree three days prior and accidentally hit his right arm against a big rock at the base of that tree. He initially felt pain in his fingers, which was relieved by putting ice-cold packs, but two days later, he noticed a change in the color of his fingers to purple so he decided to come to the ED. In the ED, his vital signs showed elevated blood pressure (160/94 mmHg), normal heart rate (70 bpm), normal pulse oxygenation level (97% on room air), and normal body temperature. His physical examination revealed well-demarcated purple tips of the right hand's second and thirds digits distal to the distal interphalangeal (DIP) joints (Figure 1) and decreased pulses of the right radial artery and right dorsalis pedis; otherwise, his exam was unremarkable. His lab workup showed a normal complete blood count (CBC) except for mild leukocytosis (12.6 K/cumm) and elevated creatinine (1.48 mg/dl). Thrombophilia and coagulation studies showed normal active partial thromboplastin time (aPTT), slightly elevated prothrombin time (PT) at 12.3 sec (normal range 9.4-11.2 sec), borderline high international normalized ratio (INR) at 1.2 (normal range 0.9-1.13), normal protein S activity, borderline low protein C activity at 64% (normal range 73-144%), normal antithrombin III antigen, absence of Factor V Leiden mutation, negative anticardiolipin antibodies, lupus anticoagulant, and beta-2-glycoprotein antibodies. His serology showed weakly positive antinuclear antibodies (ANA) with a titer of 1:80 but negative for anti-double strand DNA (dsDNA), rheumatoid factor (RF), anti-neutrophil cytoplasmic antibodies (ANCA), complement C3 and C4, erythrocyte sedimentation rate (ESR), and C-reactive protein (CRP).

**Figure 2:**
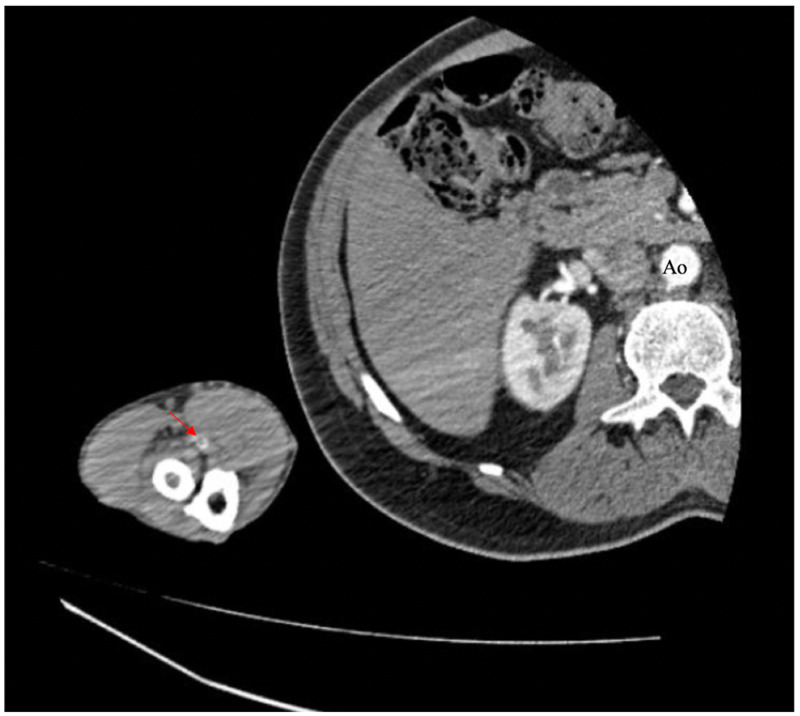
Computed tomography angiography (CTA) of the right-hand revealing thrombus formation (red arrow) and filling defect as compared to the aorta (Ao)

**Figure 3:**
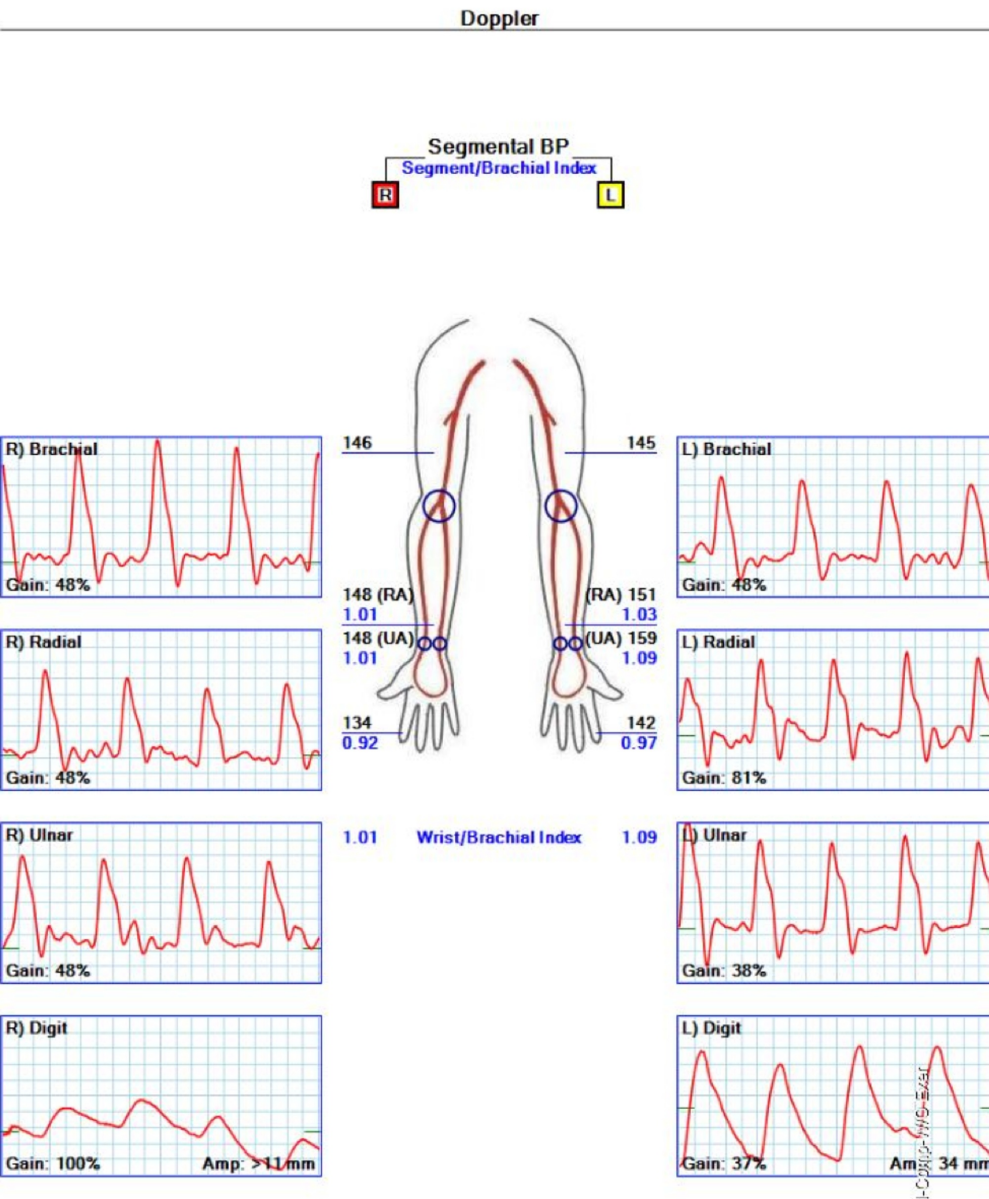
Segmental blood pressure and digital/brachial indexes

**Figure 4:**
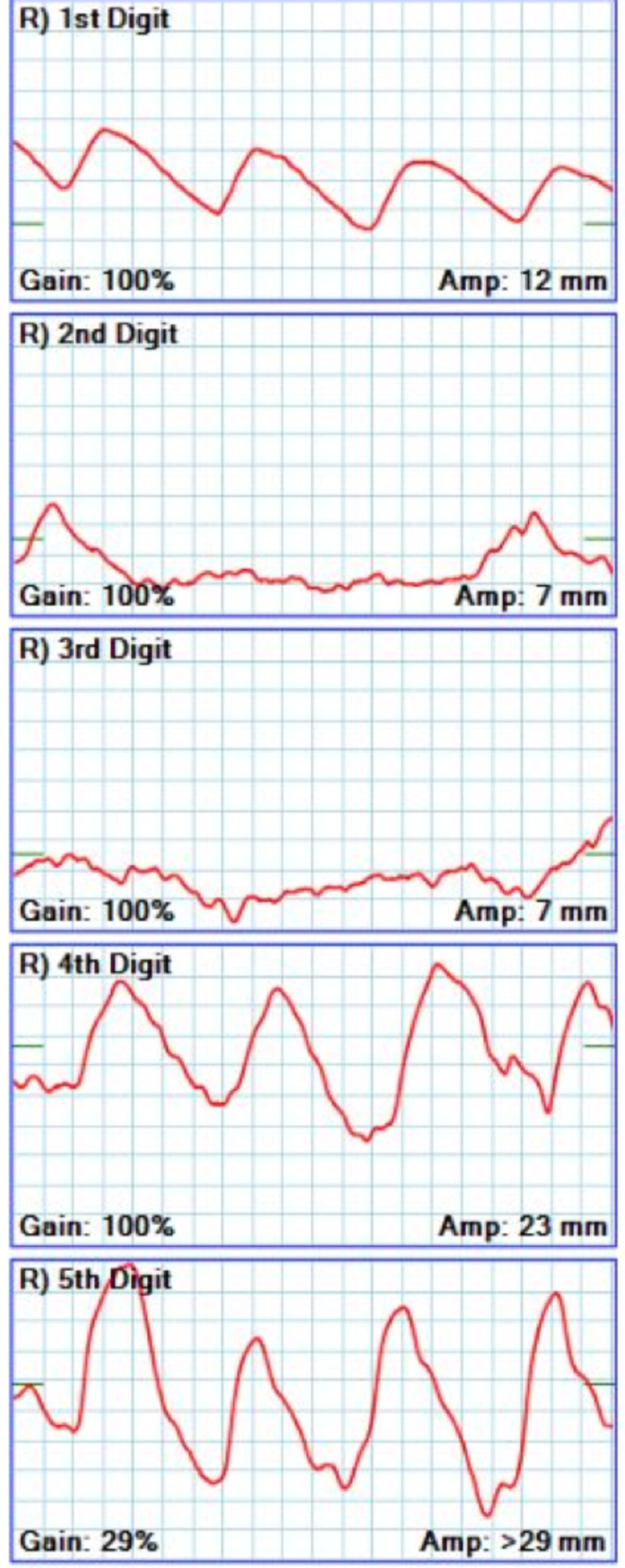
Right-hand Doppler blood flow waveform with normal triphasic waves in the first, fourth, and fifth digits and flat line in the second and third digits

Other diagnostic studies included normal electrocardiogram and transthoracic echocardiogram, without evidence of arrhythmias or cardiac thrombi. Computed tomography angiography (CTA) of the right upper extremity revealed short segment intraluminal thrombus 2 cm below the elbow joint within the right brachial artery, with diffuse vasospasm distally (Figure 2). Arterial Doppler study showed no flow to the distal second and third digits of the right hand (Figures 3-4). Due to a decrease in the right dorsalis pedis pulse, CTA of the abdomen, pelvis, and lower extremities was done and showed normal aorta and major branches, with mild atherosclerotic calcification in the femoral artery and occlusion of the anterior tibial artery at its origin.

Management

The patient was admitted to the hospital and started on aspirin and statin and was given an intravenous heparin drip for four days. After the aforementioned extensive workup that showed negative findings for systemic vasculitis or prothrombotic state, the patient was treated with nifedipine, followed by right stellate sympathetic ganglion block on days five and nine. Throughout the hospital stay, the patient continued to have a palpable radial pulse and Dopplerable ulnar signals and palmar arch signals. He was discharged home on the 13th day with aspirin, statin, and Xarelto.

## Discussion

Our case highlights the rare possibility of developing arterial thrombus after trauma, which mimics vasculitis. Although venous thromboembolism and deep venous thrombosis are known complications after trauma, arterial thrombus after trauma is rarely reported in the literature [5]. Initially, in this case, the cause of thrombosis was thought to be vasculitis but with further laboratory testing and imaging, vasculitis was ruled out. Although the patient had weakly positive ANA (titer 1:80) the rest of the workup, including serologic and inflammatory markers and complement levels, was normal, making vasculitis less likely. Other potential causes of thrombus, such as cardioembolism, were ruled out since there was no evidence of arrhythmia or intracardiac thrombus on electrocardiogram and echocardiogram, respectively. Arterial imaging revealed no evidence of atherosclerosis, and hypercoagulability was ruled out as well. Although any form of vasculitis, whether large, medium, or small-vessel vasculitis, can present with digital ischemia and gangrene, the lone manifestation of ischemia and gangrene is uncommon [6].

The diagnosis of vasculitis, in general, is challenging since the differential diagnosis is broad and it can have various manifestations. Vasculitis mimickers must be excluded first. Infections such as hepatitis B and C, human immunodeficiency virus (HIV), and tuberculosis (TB) are important secondary causes of vasculitis; therefore, they must be excluded [7-9]. Hematological malignancies, such as myeloproliferative and lymphoproliferative disorders, solid tumors, and drugs, should be ruled out. A thorough history, physical examination, and a battery of laboratory testing and imaging can all help in differentiating one from another [10].

Post-traumatic venous thromboembolism and deep venous thrombosis have been well-described in the literature but arterial thrombosis after trauma is a rare entity [11]. The mechanism of endothelial injury for arterial thrombosis differs from that of venous thrombosis. Underlying atherosclerosis or hypertension, which creates turbulence in the blood flow, ultimately resulting in the activation of platelets and the formation of a plaque, is thought to play a key role in arterial thrombus formation [12].

To the best of our knowledge, this is the first case in the literature that presented with a vasculitis-like picture after trauma. Karaarslan et al. have reported a case of ulnar arterial thrombosis after a trauma that presented with hand pain and swelling rather than digital gangrene [13]. “Hypothenar-hammer” syndrome has been commonly described in the literature, which refers to an injury to the ulnar artery as a consequence of repetitive trauma, which results in thrombosis and embolism of digital arteries. It is most commonly seen in athletes (hockey players, karate experts) and some occupations such as machinery, carpentry, or any industry requiring repeated striking of tools. However, arterial thrombosis after single blunt trauma is rarely described [14-15].

## Conclusions

Post-traumatic arterial thrombosis are uncommon, and their clinical presentation can mimic systemic vasculitis. Vasculitis mimickers have been reported and usually create a real conundrum to clinicians. In the context of arterial thrombosis with gangrene, it is crucial to consider and rule out other etiologies, including systemic vasculitis, as the therapeutic approach varies widely and a misdiagnosis can lead to life-threatening outcomes. Therefore, vasculitis mimickers should also be ruled out as a potential cause of gangrene.
